# Practical problems may preclude realization of this proposal

**DOI:** 10.2196/jmir.2.3.e15

**Published:** 2000-09-12

**Authors:** Vincenzo Della Mea

## Commentary

The topic discussed in the paper of James Till [1] is very interesting and urgent, now that medicine has also joined the preprint era with other scientific fields.

The author suggests that journals may use the preprints archives as a source for finding papers to be published, going into competition to obtain the best papers available, in the manner already outlined a year ago by other authors [2]. At a first glance, it is an intriguing proposal mainly from the author's point of view; the author could wait for some journal to offer publication instead of actively submitting his or her paper.

However, there is an issue that is not very clear in light of the current discussions about preprints and their relationship to the financial issues of publishing.

First of all, if preprints become successful, it can be supposed that there will be a very large number of them, comparable to the numbers of the physics archive ArXiv [3] which are distributed onto many different servers. Thus, it is arguable that the task of discovering interesting preprints will add significantly to the workload to be carried out on the editor/publisher side. Furthermore, this additional filtering work might be, in principle, harder on editors than traditional peer review, because the latter is based on some form of auto-filtering by the authors themselves who already send papers to the "right" journals. Although usually referees work for free, preprint selection from a large document base may incur some additional costs for publishers, who already fear income shortenings from the birth of publicly accessible archives.

Author fees have been proposed as a solution to financially sustain traditional journals, representing the added value they provide (i.e., the sort of "quality stamp" given by peer review, editing, and diffusion) [4] [5] in the new model of free scientific communication. This approach will shift costs from readers to authors, opening research results to a wider audience, and at the same time letting traditional publishers survive. However, the author fees issue leads to another unclear question not answered in the paper. The author mentions the competition among editors for publishing interesting preprints. How will this competition evolve in an model in which authors are paying for the right to be published? I suppose that the competition could include the reduction of author fees for exceptional articles, but perhaps only journals with other funding resources could manage to afford that cost. In addition, some form of author payment might be introduced, as already proposed by the BMJ [6], to increase the journal's attractiveness.

Just to summarize, I suspect that journals would need to adopt different organization models to inact to the author's proposal (e.g., employ new article seekers in addition to referees, and so on) with associated higher costs, and competition for articles may include economic aspects in conflict with the expected page charge that will likely be used to cover publication expenses when papers will be freely available online. So, although the idea is interesting, I'm not sure how it can practically be adopted, and I simply would like to see some discussion about this.

Another point worthy of discussion concerns comments and responses to Netprints. Why so few?

Nobody works for free: comments and responses, to be useful, should be as accurate as the usual (good) referee comments, which are work in exchange for prestige. In the same way, letters provide useful comments to authors (although after publication), but are usually regarded as small publications useful for the letter writer's resume, above all when appearing in prestigious journals.

Once it is recognized that comments and responses to preprints (and generally to online documents) are useful for improving science, it might be possible to solicit comments by providing the senders of responses that enhance the quality of the paper with an acknowledgement as an incentive, similar to a junior authorship. The mechanisms to enable such an incentive would be very difficult to evaluate and implement; however, there exists a germinal proposal [7] that links the comment activity to a specific, automatically calculated personal value to be added to something similar to a personal impact factor (which would derive from the comments).

Finally, I completely agree on the need for evaluative studies of preprints. Since the ClinMed NetPrints archive are still in the early stages, I wonder if there is a study already in existence on the effect of preprints in physics and their relationship to the publication process. Such an analysis could give very effective advice on how medical preprints can be used to improve medical science.

## References

[1] Till J E (2000). Peer review in a post-eprints world: a proposal. J Med Internet Res.

[2] Allan B, Morlet N, Wormald R (1997). Journals and the Internet. Use of the Internet for on line peer review must be explored further. BMJ.

[3] The arXiv Archives.

[4] Varmus H E-Biomed: a proposal for electronic publications in the biomedical sciences.

[5] Harnad S (1998). On-line journals and financial fire walls. Nature.

[6] Smith J (2000). Giving something back to authors. Some changes to our copyright agreements. BMJ.

[7] Mizzaro S (2000). An Automatically Refereed Scholarly Electronic Journal. Proceedings of the MEIS'2000 - Media in Information Society Conference.

